# Elevated inflammatory index and future risk of stroke in patients with coronary heart disease: a multicenter prospective cohort study

**DOI:** 10.3389/fcvm.2025.1755408

**Published:** 2026-01-12

**Authors:** Jie Cheng, Keli Li, Meng Chen, Yan Yu, Zhaowang Tan

**Affiliations:** 1 Department of Cardiology II and Geriatric Management Ward, Zhangye Second People's Hospital, Zhangye, China; 2 Department of General Medicine of Huangshan City People's Hospital, Huangshan, China; 3 Department of Cardiology, Suzhou Hospital of Anhui Medical University, Suzhou, China; 4 Emergency and Critical Care Center, Department of Emergency Medicine, Zhejiang Provincial People's Hospital (Affiliated People's Hospital), Hangzhou Medical College, Hangzhou, Zhejiang, China

**Keywords:** coronary heart disease, hemorrhagic stroke, inflammatory markers, ischemic stroke, stroke

## Abstract

**Background:**

Previous studies have consistently shown that patients with coronary heart disease (CHD) face an elevated risk of stroke, with inflammation likely playing a significant contributing role. Building on this evidence, the present study aims to investigate the associations between three emerging inflammatory markers and the risk of stroke in patients with CHD, as well as to identify which marker offers the greatest clinical predictive value.

**Methods:**

This study involved 5,289 patients with CHD from three clinical centers. COX regression analysis was conducted to examine the relationships between three inflammatory markers—systemic inflammatory response index (SIRI), systemic immune-inflammation index (SII), and aggregate index of systemic inflammation (AISI)—and the risk of stroke in patients with CHD, and Kaplan–Meier (KM) curves were generated to visualize stroke incidence across different marker levels. In addition, restricted cubic splines (RCS) were applied to assess potential dose–response relationships. Finally, receiver operating characteristic curves and C-statistics were used to evaluate the predictive performance of the three inflammatory markers for stroke risk.

**Results:**

During the 4.82-year median follow-up period, a total of 785 participants experienced stroke events. The COX regression analysis showed that all three inflammatory markers were significantly associated with an increased risk of stroke in patients with CHD. Consistently, the KM curves indicated that patients with higher levels of inflammation experienced a markedly higher incidence of stroke compared with those in the low-inflammation group. Furthermore, the RCS analysis revealed that when AISI, SII, and SIRI exceeded 117, 450, and 0.95, respectively, the risk of stroke rose substantially. Finally, the comparative analysis showed that AISI demonstrates significantly better predictive performance.

**Conclusion:**

These three inflammatory indicators are closely linked to an increased risk of stroke in patients with CHD. In addition, comparative analysis indicates that the inflammatory index represented by AISI has superior predictive performance. Consequently, AISI may offer a feasible tool for early monitoring and risk assessment of stroke in patients with CHD.

## 1 Introduction

Stroke is a severe cerebrovascular disease characterized by extremely high mortality and disability rates (1–3). In recent years, the growing prevalence of chronic conditions such as hypertension, obesity, diabetes, and coronary heart disease (CHD) has contributed to a marked increase in stroke incidence (1, 2, 4). Statistical reports show that between 1990 and 2019, there were 12.2 million new stroke cases, 101 million individuals living with stroke, 1.43 billion disability-adjusted life years lost, and 6.55 million deaths attributable to stroke (4–6). Among older adults in particular, CHD frequently coexists with stroke, and previous studies have demonstrated that patients with CHD are at a higher risk of developing stroke (7, 8). This comorbidity further worsens clinical outcomes in this population and places substantial economic and caregiving burdens on families and society. Therefore, early identification of stroke risk in patients with CHD, along with timely intervention, is especially critical.

Compared with traditional studies that primarily attributed stroke and cardiovascular death in patients with CHD to suboptimal control of blood pressure, blood glucose, and blood lipids, as well as to obesity-associated metabolic abnormalities, the role of inflammation has received considerably less attention (9–11). However, accumulating evidence in recent years indicates that both inflammation and residual inflammation play critical roles in elevating stroke risk among patients with CHD (12–15). Atherosclerosis in these patients begins with the accumulation of oxidized low-density lipoprotein in the arterial wall, which stimulates endothelial cells to produce pro-inflammatory molecules and recruit inflammatory cells and chemokines (14, 16–18). This process initiates an inflammatory cascade that damages the vascular wall and promotes thrombosis (16, 19). Simultaneously, inflammatory cells contribute to collagen degradation, fibrous cap erosion, tissue factor release, and the activation of platelets and coagulation pathways, ultimately leading to thromboembolic events (13, 20, 21). These physiological and pathological mechanisms collectively demonstrate that inflammation is central to the initiation, progression, erosion, and rupture of atherosclerotic plaques (16, 22). Furthermore, several studies have repeatedly shown that effective inflammation control can reduce the risk of stroke (9, 12, 13). Taken together, these findings underscore the pivotal contribution of inflammation to stroke risk in patients with CHD.

Given that inflammation often involves multiple indicators—such as white blood cells, neutrophils, monocytes, lymphocytes, and C-reactive protein—relying on a single indicator may fail to truly reflect the immune-inflammatory state of the body. As a result, researchers have developed simple and easily obtainable composite inflammatory indicators derived from blood cell counts (23–25). For example, the Systemic Immune Inflammation Index (SII), the Systemic Inflammatory Response Index (SIRI), and the aggregate index of systemic inflammation (AISI) have begun to attract attention (25, 26). These indices combine multiple blood cell parameters and can more accurately reflect the body's inflammatory condition. Moreover, they have demonstrated strong predictive performance across various diseases (24, 25, 27). For instance, in patients with fatty liver disease associated with hypertension, these inflammatory indicators have shown superior predictive ability (25). Similarly, in some patients with sepsis, indicators like SIRI have been particularly effective at predicting mortality risk (27). Collectively, these studies provide compelling evidence for the broader adoption of these new inflammatory indicators.

Given the important role of inflammation in the development of both CHD and stroke, as well as the strong predictive performance of these inflammatory markers across various diseases, this study aims to investigate the associations between these emerging inflammatory indices and the risk of stroke in patients with CHD. In addition, the study seeks to identify the best inflammatory marker, which may provide a basis for future stroke risk assessment and early intervention in patients with CHD.

## 2 Material and methods

### 2.1 Study population

**Figure 1:**
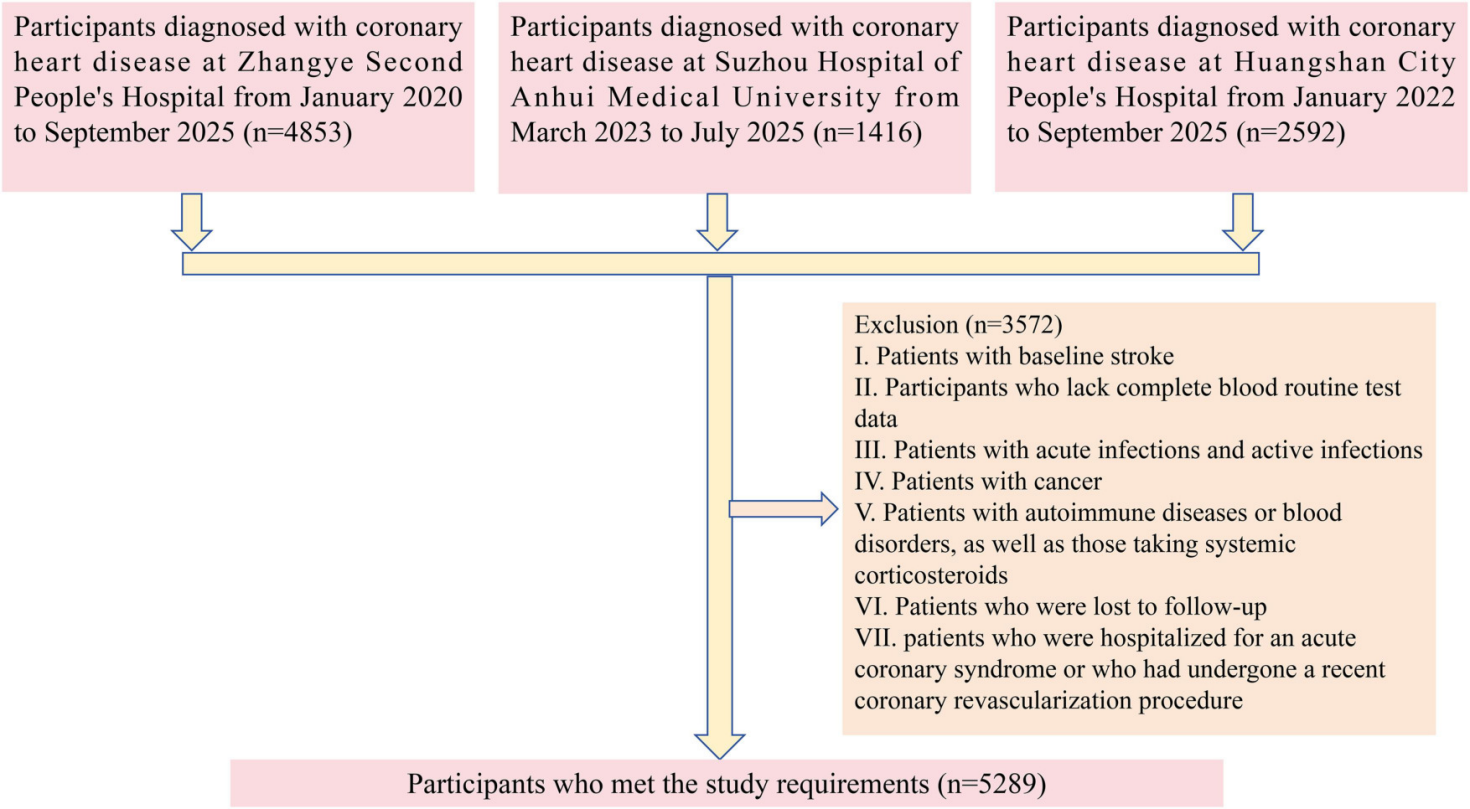
Flowchart illustrating the selection of the study population.

We collected relevant data from a total of 8,861 patients with CHD across three medical centers, as detailed below. From January 2020 to September 2025, hospitalized patients diagnosed with CHD at Zhangye Second People's Hospital were included. From March 2023 to July 2025, hospitalized patients diagnosed with CHD at Suzhou Hospital of Anhui Medical University were included. From January 2022 to September 2025, hospitalized patients diagnosed with CHD at Huangshan City People's Hospital were included. Exclusion criteria were as follows: (1) prior diagnosis of stroke; (2) incomplete blood test results; (3) presence of tumors; (4) autoimmune or hematologic diseases; (5) loss to follow-up during the collection period; (6) patients who were hospitalized for an acute coronary syndrome or who had undergone a recent coronary revascularization procedure; (7) patients with active infections or those receiving systemic corticosteroid therapy at the time of enrollment. Figure 1 illustrates the specific inclusion and exclusion process.

This study complied with the Helsinki Declaration and received approval from the ethics committees of Zhangye Second People's Hospital (ZYEY201911), Suzhou Hospital of Anhui Medical University (SZ.N.202211), and Huangshan City People's Hospital (HS202106). All participants provided written informed consent prior to enrollment.

### 2.2 Data collection

This observational study collected data across four main domains. First, demographic characteristics were recorded, including sex, age, body mass index (BMI), systolic and diastolic blood pressure, smoking status, and alcohol consumption. Second, laboratory parameters were obtained, comprising routine blood tests, alanine aminotransferase (ALT), aspartate aminotransferase (AST), total cholesterol, triglycerides, high-density lipoprotein cholesterol (HDL-C), low-density lipoprotein cholesterol (LDL-C), and fasting plasma glucose (FPG). Third, medical history was assessed, with particular attention to diabetes, hypertension, and disorders of lipid metabolism. Finally, information on current medications was collected, including statins, antiplatelet agents, beta-blockers, and angiotensin-converting enzyme inhibitors (ACEIs) or angiotensin receptor blockers (ARBs). All blood samples were obtained from routine admission tests after patients' clinical conditions had stabilized and were collected prior to any elective surgery or interventional procedures.

The inflammatory indices AISI, SII, and SIRI were calculated using the following formulas (23, 25, 27): AISI = (neutrophil count × monocyte count ×  platelet count)/lymphocyte count; SII = (platelet count  ×  neutrophil count)/lymphocyte count; and SIRI = (neutrophil count  ×  monocyte count)/lymphocyte count.

### 2.3 Study outcome

The primary outcome for this study was the incident stroke (including ischemic stroke and hemorrhagic stroke). The secondary outcomes for this study were ischemic stroke and hemorrhagic stroke, respectively. Data on endpoint events were collected through multiple channels, including medical visit records, medical insurance data, telephone follow-ups, and regional disease and death registration offices. Patients were followed from the time of enrollment until the end of the study, which was defined as the date of the last follow-up appointment, the date of the first observed stroke, the date of death, or the date when the investigation was completed.

### 2.4 Statistical analysis

Participants were classified into either the stroke group or the non-stroke group based on clinical outcomes. Continuous variables were summarized as mean ± standard deviation or median with interquartile range, according to data distribution, whereas categorical variables were presented as frequencies and percentages.

Regarding the handling of missing data, for variables with less than 20% missing values, we performed multiple imputation using the “mice” package in R. Prior to adjustment, a multicollinearity diagnostic was conducted, which confirmed no significant multicollinearity among the variables (Supplementary Table S1). Additionally, the proportional hazards assumption was assessed before the main analysis and was found to be satisfied (Supplementary Figure S1).

The inflammatory indices AISI, SII, and SIRI were each categorized into tertiles. Cox proportional hazards regression models were used to evaluate the associations between these indices and the risk of stroke in patients with CHD. To assess the robustness of the findings, a series of models with progressive adjustment for demographic factors, laboratory parameters, and medical history were constructed. Kaplan–Meier (KM) curves were plotted to compare event-free survival across tertile groups of AISI, SII, and SIRI. Restricted cubic spline (RCS) analyses were performed to explore potential dose–response relationships between these inflammatory markers and stroke risk, with turning points determined from the spline curves. Logistic regression analysis was applied to construct receiver operating characteristic (ROC) curves, and C statistics were calculated to evaluate the predictive performance of AISI, SII, and SIRI for overall stroke and its subtypes. Detailed statistical procedures are provided in the Supplementary Materials.

Statistical significance was defined as a *p*-value <0.05. All statistical analyses were performed using R software (version 4.4.3).

## 3 Results

### 3.1 Basic characteristics of participants

Over a median follow-up period of 4.82 (2.21, 7.24) years, 785 participants experienced a stroke, corresponding to an incidence rate of 31 events per 1,000 person-years. Compared with the non-stroke group, patients who developed stroke were older, had a higher proportion of males (80.38%), and exhibited a higher BMI. In terms of laboratory findings, the stroke group showed significantly higher levels of ALT, AST, triglycerides, total cholesterol, LDL-C, FPG, as well as the inflammatory indices AISI, SII, and SIRI, while HDL-C levels were lower. With respect to medical history, the prevalence of hypertension (74.52%) and dyslipidemia (62.55%) was significantly greater in the stroke group. Regarding medication use, patients in the stroke group had lower rates of statin, antiplatelet, and beta-blocker use. All of these differences were statistically significant. In contrast, no significant differences were observed between the two groups in the use of ACEIs or ARBs, the prevalence of diabetes, or baseline blood pressure levels (Table 1).

**Table 1:** Baseline characteristics of the study population.

Characteristic	Overall	Non-Stroke	Stroke	*p* value
*N*	5,289	4,504	785	
Age (years)	58.47 ± 8.26	57.73 ± 7.90	58.60 ± 8.31	0.007
Sex (%)				<0.001
Female	1,496 (28.29%)	1,342 (29.80%)	154 (19.62%)	
Male	3,793 (71.71%)	3,162 (70.20%)	631 (80.38%)	
BMI (kg/m^2^)	25.75 ± 3.97	25.64 ± 3.96	26.40 ± 3.94	<0.001
SBP (mmHg)	145.45 ± 18.40	145.38 ± 18.39	145.83 ± 18.51	0.535
DBP (mmHg)	87.89 ± 13.52	87.75 ± 13.32	88.69 ± 14.60	0.071
Smoking (%)	725 (13.71%)	567 (12.59%)	158 (20.13%)	
Drinking (%)	531 (10.04%)	399 (8.86%)	132 (16.82%)	
Laboratory tests				
ALT (U/L)	17.30 (12.00–28.00)	17.00 (12.00–27.42)	20.97 (14.00–31.00)	<0.001
AST (U/L)	18.33 (15.00–24.00)	18.00 (15.00–24.00)	19.30 (16.00–26.00)	0.001
TC (mmol/L)	4.10 ± 0.93	4.09 ± 0.92	4.18 ± 0.98	0.013
TG (mmol/L)	0.66 (0.55–1.47)	0.65 (0.55–1.38)	0.70 (0.58–1.99)	<0.001
HDL-C (mg/dL)	1.17 ± 0.28	1.18 ± 0.28	1.09 ± 0.28	<0.001
LDL-C (mg/dL)	2.74 ± 0.80	2.67 ± 0.79	3.15 ± 0.76	<0.001
FPG (mmol/L)	4.74 ± 0.90	4.72 ± 0.92	4.89 ± 0.76	<0.001
AISI	116.65 (80.71–161.95)	111.20 (78.51–152.64)	168.37 (111.52–265.95)	<0.001
SII	453.42 (328.40–612.91)	435.54 (319.14–581.81)	603.95 (421.98–827.88)	<0.001
SIRI	0.95 (0.75–1.23)	0.92 (0.73–1.17)	1.17 (0.88–1.58)	<0.001
Medical history				
DM	817 (15.54%)	667 (14.81%)	150 (19.11%)	0.088
Hypertension	2,980 (52.56%)	2,195 (48.73%)	585 (74.52%)	<0.001
Dyslipidemia	2,494 (47.15%)	2,003 (44.47%)	491 (62.55%)	<0.001
Medications				
Statins (%)	5,260 (99.45%)	4,494 (99.78%)	766 (97.58%)	<0.001
Antiplatelet medication (%)	5,205 (98.41%)	4,474 (99.33%)	731 (93.12%)	<0.001
Beta-blockers (%)	5,110 (96.62%)	4,381 (97.27%)	729 (92.87%)	<0.001
ACEIs/ARBs	4,607 (87.11%)	3,910 (86.81%)	697 (88.79%)	0.127

Data are presented as mean ± standard deviation, median (interquartile range), or as numbers, and percentages.

ACEIs, angiotensin-converting enzyme inhibitors; AISI, aggregate index of systemic inflammation; ALT, alanine transaminase; ARBs, angiotensin receptor blockers; AST, aspartate transaminase; BMI, body mass index; DBP, diastolic blood pressure; DM, diabetes mellitus; FPG, fasting plasma glucose; HDL-C, high-density lipoprotein cholesterol; LDL-C, low-density lipoprotein cholesterol; SBP, systolic blood pressure; SII, Systemic Immune-Inflammation Index; SIRI, Systemic Inflammation Response Index; TC, total cholesterol; TG, triglyceride.

### 3.2 Relationship between inflammatory markers and stroke in patients with CHD

**Figure 2:**
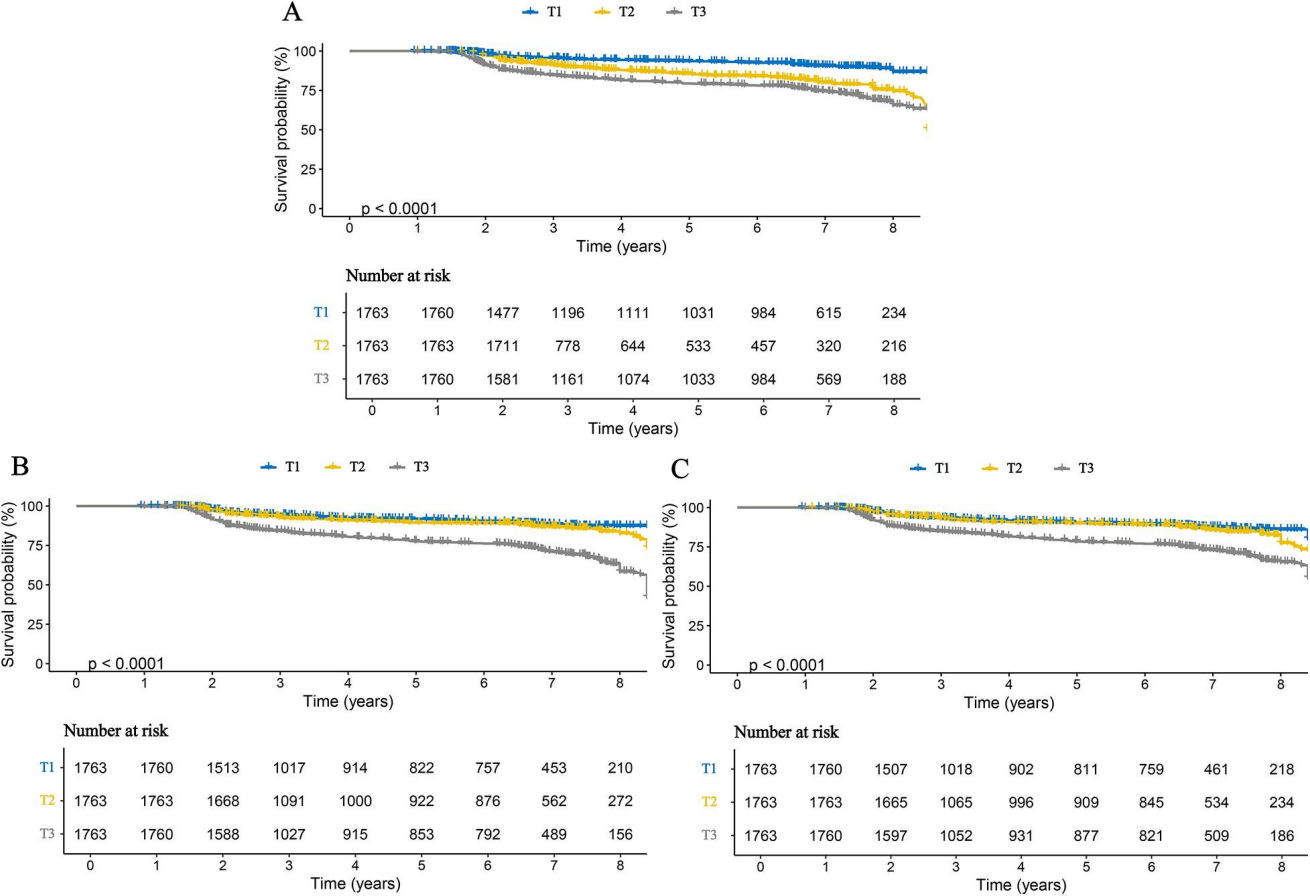
Kaplan–Meier curves for stroke in patients with CHD, stratified by tertiles of three inflammatory markers. **(A)** AISI; **(B)** SII; **(C)** SIRI.

Cox proportional hazards regression analysis showed that all three inflammatory markers were significantly associated with an increased risk of stroke in patients with CHD.In the fully adjusted Model 4, each one standard deviation (SD) increase in AISI, SII, and SIRI was associated with hazard ratios (HRs) of 2.914 (95% confidence interval [CI]: 2.668–3.182), 2.007 (95% CI: 1.819–2.213), and 2.817 (95% CI: 2.450–3.224), respectively (Table 2). When analyzed as categorical variables based on tertiles, these associations remained significant. Compared with the T1 group (lowest inflammatory level), participants in the T2 and T3 groups had progressively higher risks of stroke, demonstrating a significant dose–response trend (*p* for trend <0.001) (Table 2). Consistently, KM curves indicated that the cumulative incidence of stroke was significantly higher in the T2 and T3 groups than in the T1 group throughout the follow-up period (Figure 2). Furthermore, analyses conducted in the validation cohort from Huangshan City People's Hospital yielded similar findings, confirming that stroke risk increased in parallel with higher levels of inflammatory markers (Supplementary Table S2).

**Table 2:** Relationship between the inflammatory index of patients with CHD and stroke.

Stroke	Model 1	Model 2	Model 3	Model 4
	HR (95% CI) *p*	HR (95% CI) *p*	HR (95% CI) *p*	HR (95% CI) *p*
AISI				
AISI (per 1SD increase)	3.322 [3.087, 3.576] < 0.001	3.014 [2.789, 3.257] < 0.001	2.981 [2.747, 3.236] < 0.001	2.914 [2.668, 3.182] < 0.001
Tertiles of AISI				
Tertile 1	Reference	Reference	Reference	Reference
Tertile 2	3.21 [2.548, 4.054] < 0.001	2.000 [1.612, 2.482] < 0.001	1.810 [1.456, 2.249] < 0.001	2.160 [1.741, 2.680] < 0.001
Tertile 3	4.697 [3.786, 5.828] < 0.001	3.204 [2.631, 3.901] < 0.001	2.975 [2.441, 3.625] < 0.001	3.260 [2.680, 3.967] < 0.001
*p* for trend	<0.001	<0.001	<0.001	<0.001
SII				
SII (per 1SD increase)	2.528 [2.313, 2.763] < 0.001	2.410 [2.202, 2.637] < 0.001	2.474 [2.254, 2.716] < 0.001	2.007 [1.819, 2.213] < 0.001
Tertiles of SII				
Tertile 1	Reference	Reference	Reference	Reference
Tertile 2	1.567 [1.253, 1.961] < 0.001	1.260 [1.014, 1.567] < 0.001	1.303 [1.046, 1.624] < 0.001	1.287 [1.036, 1.598] < 0.001
Tertile 3	2.851 [2.329, 3.491] < 0.001	3.131 [2.585, 3.791] < 0.001	3.119 [2.570, 3.785] < 0.001	3.197 [2.641, 3.871] < 0.001
*p* for trend	<0.001	<0.001	<0.001	<0.001
SIRI				
SIRI (per 1SD increase)	3.753 [3.306, 4.259] < 0.001	3.451 [3.037, 3.921] < 0.001	3.515 [3.078, 4.015] < 0.001	2.817 [2.450, 3.224] < 0.001
Tertiles of SIRI				
Tertile 1	Reference	Reference	Reference	Reference
Tertile 2	1.270 [1.033, 1.563] 0.023	1.183 [0.961, 1.457] 0.113	1.157 [0.938, 1.426] 0.173	1.393 [1.128, 1.721] 0.002
Tertile 3	2.585 [2.150, 3.109] < 0.001	2.536 [2.108, 3.052] < 0.001	2.390 [1.984, 2.880] < 0.001	2.309 [1.897, 2.811] < 0.001
*p* for trend	<0.001	<0.001	<0.001	<0.001

Model 1: no covariates were adjusted.

Model 2: age, sex, BMI, smoking status and drinking status were adjusted.

Model 3: Model 2 plus adjustment for SBP, DBP, TC, TG, HDL.C, LDL.C, and FPG.

Model 4: Model 3 plus adjustment for Statins, Antiplatelet medication, Beta-blockers, DM, Dyslipidemia and Hypertension.

AISI, aggregate index of systemic inflammation; CI, confidence interval; HR, hazard ratio; SII, Systemic Immune-Inflammation Index; SIRI, Systemic Inflammation Response Index.

Other abbreviations, see Table 1.

### 3.3 Relationship between inflammatory markers and stroke subtypes in patients with CHD

**Figure 3:**
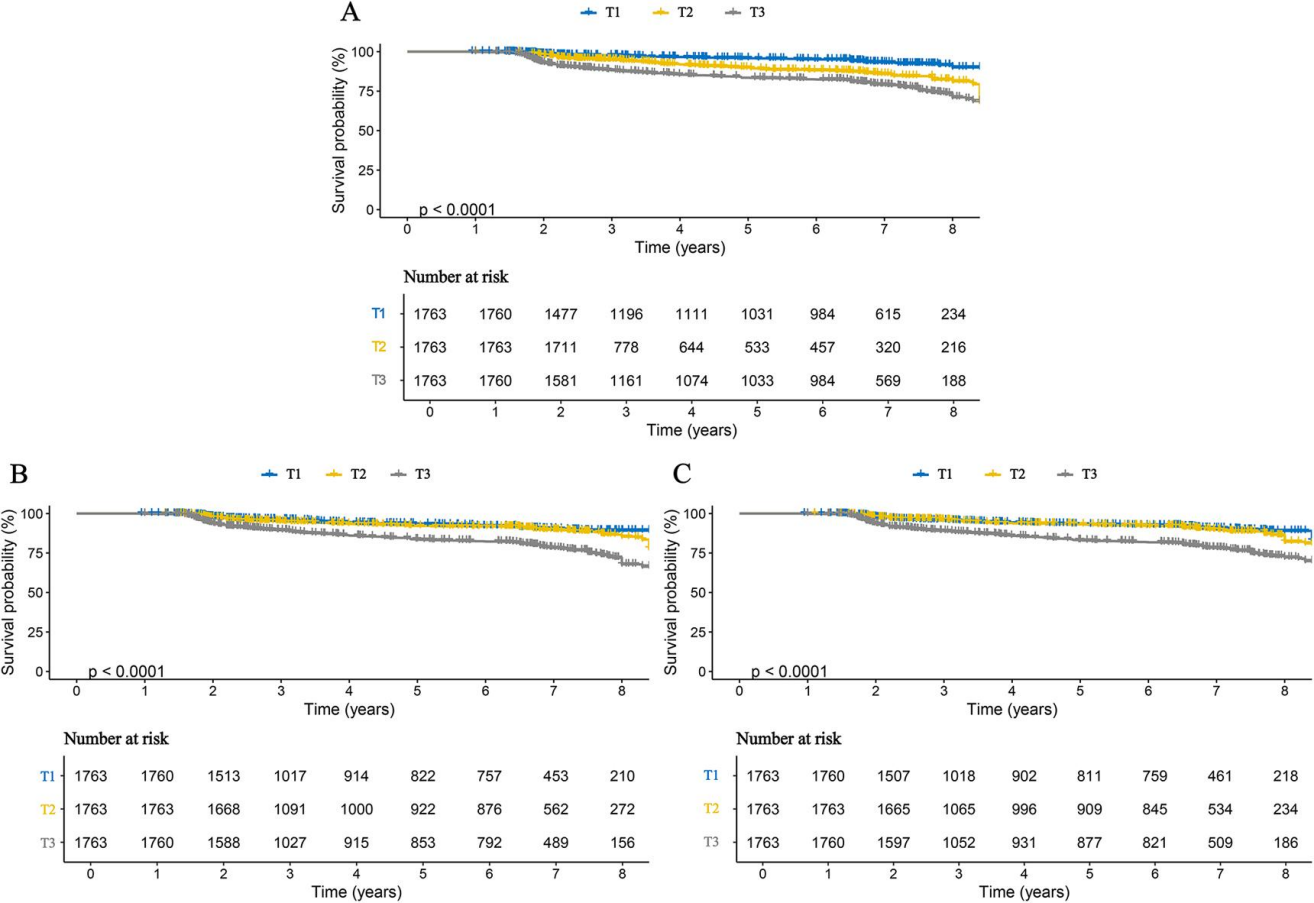
Kaplan–Meier curves for ischemic stroke in patients with CHD, stratified by tertiles of three inflammatory markers. **(A)** AISI; **(B)** SII; **(C)** SIRI.

**Figure 4:**
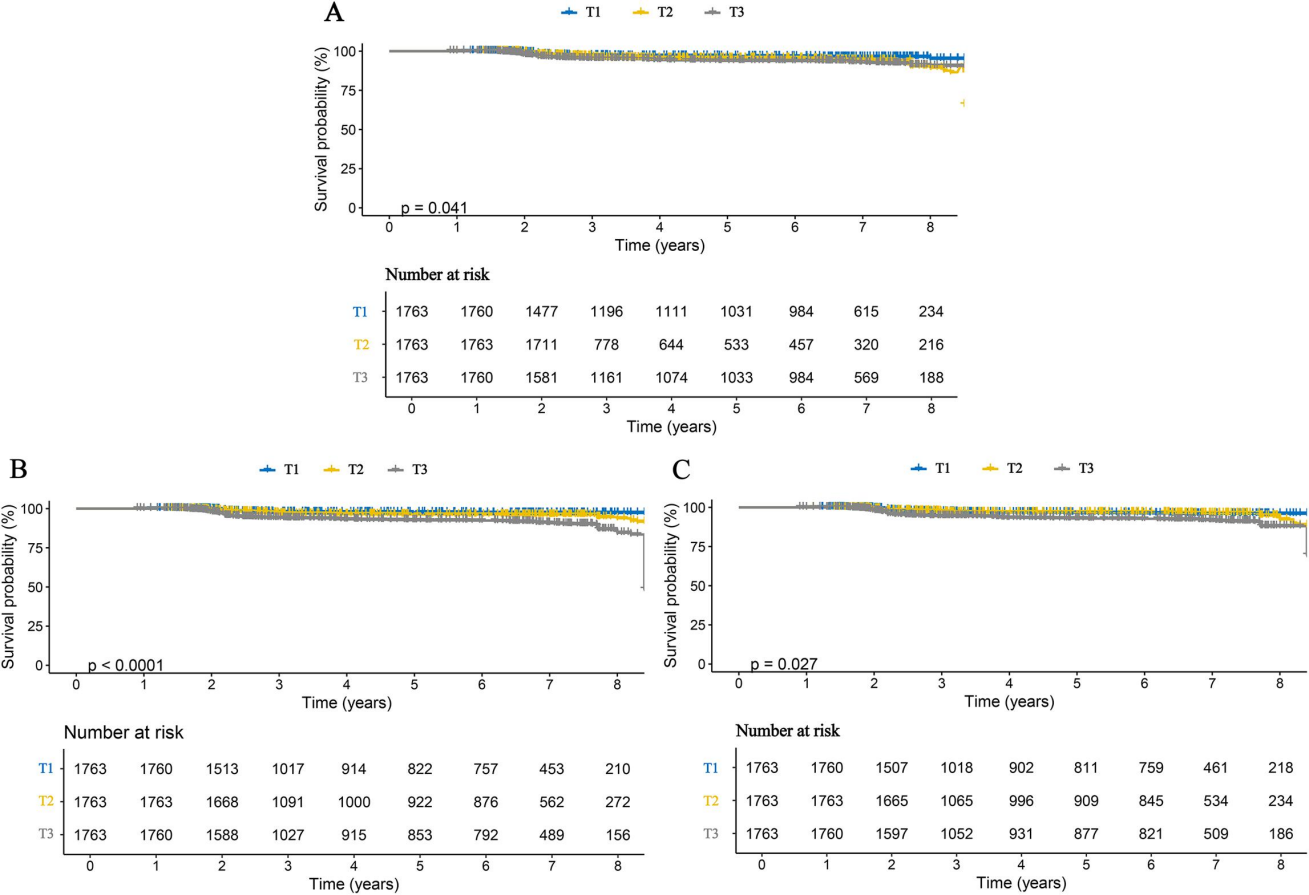
Kaplan–Meier curves for hemorrhagic stroke in patients with CHD, stratified by tertiles of three inflammatory markers. **(A)** AISI; **(B)** SII; **(C)** SIRI.

We further examined the associations between inflammatory markers and specific stroke subtypes. The results were consistent with those observed for overall stroke and remained robust for both ischemic and hemorrhagic stroke. Specifically, each one SD increase in AISI, SII, and SIRI was associated with a 3.111-fold (95% CI: 2.816–3.438), 1.947-fold (95% CI: 1.737–2.181), and 2.938-fold (95% CI: 2.510–3.436) higher risk of ischemic stroke, respectively. Correspondingly, the risks of hemorrhagic stroke increased by 2.660-fold (95% CI: 2.249–3.147), 2.487-fold (95% CI: 2.099–2.947), and 3.061-fold (95% CI: 2.392–3.916) for each SD increase in AISI, SII, and SIRI, respectively (Table 3). When these inflammatory markers were analyzed as categorical variables, higher tertile levels were associated with progressively increased risks of both ischemic and hemorrhagic stroke, indicating clear dose–response relationships (Table 3). These findings were further corroborated by Kaplan–Meier curve analyses (Figures 3, 4). Consistent results were also observed in the validation analysis based on data from the People's Hospital of Huangshan City (Supplementary Table S3). Finally, the results of the E-value analysis indicate that these factors are very unlikely to overturn our findings (Supplementary Table S4).

**Table 3:** Relationship between inflammatory indicators in patients with CHD and the subtypes of stroke.

Stroke subtype	Model 1	Model 2	Model 3	Model 4
	HR (95% CI) *p*	HR (95% CI) *p*	HR (95% CI) *p*	HR (95% CI) *p*
Ischemic stroke				
AISI				
AISI (per 1SD increase)	3.490 [3.214, 3.790] < 0.001	3.086 [2.827, 3.369] < 0.001	3.067 [2.797, 3.362] < 0.001	3.111 [2.816, 3.438] < 0.001
Tertiles of AISI				
Tertile 1	Reference	Reference	Reference	Reference
Tertile 2	2.072 [1.600, 2.685] < 0.001	1.897 [1.465, 2.458] < 0.001	1.737 [1.338, 2.253] < 0.001	3.316 [2.516, 4.372] < 0.001
Tertile 3	3.639 [2.892, 4.579] < 0.001	3.561 [2.828, 4.484] < 0.001	3.351 [2.658, 4.224] < 0.001	5.661 [4.403, 7.278] < 0.001
*p* for trend	<0.001	<0.001	<0.001	<0.001
SII				
SII (per 1SD increase)	2.429 [2.186, 2.700] < 0.001	2.285 [2.055, 2.540] < 0.001	2.358 [2.114, 2.631] < 0.001	1.947 [1.737, 2.181] < 0.001
Tertiles of SII				
Tertile 1	Reference	Reference	Reference	Reference
Tertile 2	1.229 [0.965, 1.566] 0.095	1.208 [0.948, 1.541] 0.127	1.273 [0.996, 1.627] 0.054	1.580 [1.230, 2.028] < 0.001
Tertile 3	2.729 [2.201, 3.385] < 0.001	2.674 [2.156, 3.318] < 0.001	2.729 [2.194, 3.395] < 0.001	2.589 [2.059, 3.254] < 0.001
*p* for trend	<0.001	<0.001	<0.001	<0.001
SIRI				
SIRI (per 1SD increase)	3.868 [3.342, 4.478] < 0.001	3.445 [2.974, 3.991] < 0.001	3.530 [3.032, 4.110] < 0.001	2.938 [2.512, 3.436] < 0.001
Tertiles of SIRI				
Tertile 1	Reference	Reference	Reference	Reference
Tertile 2	1.195 [0.937, 1.522] 0.150	1.100 [0.861, 1.403] 0.446	1.084 [0.848, 1.385] 0.520	1.339 [1.046, 1.714] 0.021
Tertile 3	2.617 [2.115, 3.237] < 0.001	2.537 [2.049, 3.140] < 0.001	2.442 [1.969, 3.027] < 0.001	2.507 [1.998, 3.145] < 0.001
*p* for trend	<0.001	<0.001	<0.001	<0.001
Hemorrhagic stroke				
AISI				
AISI (per 1SD increase)	3.057 [2.651, 3.525] < 0.001	2.948 [2.542, 3.420] < 0.001	2.925 [2.495, 3.428] < 0.001	2.660 [2.249, 3.147] < 0.001
Tertiles of AISI				
Tertile 1	Reference	Reference	Reference	Reference
Tertile 2	2.152 [1.450, 3.192] < 0.001	1.881 [1.325, 2.671] < 0.001	1.823 [1.283, 2.591] < 0.001	1.600 [1.121, 2.283] 0.010
Tertile 3	2.444 [1.668, 3.581] < 0.001	2.137 [1.530, 2.984] < 0.001	2.184 [1.561, 3.057] < 0.001	1.984 [1.414, 2.783] < 0.001
*p* for trend	<0.001	<0.001	<0.001	<0.001
SII				
SII (per 1SD increase)	2.963 [2.561, 3.427] < 0.001	2.938 [2.527, 3.417] < 0.001	3.069 [2.620, 3.596] < 0.001	2.487 [2.099, 2.947] < 0.001
Tertiles of SII				
Tertile 1	Reference	Reference	Reference	Reference
Tertile 2	1.693 [1.119, 2.560] 0.013	1.662 [1.098, 2.516] 0.016	1.634 [1.075, 2.484] 0.021	1.784 [1.163, 2.738] 0.008
Tertile 3	4.197 [2.885, 6.107] < 0.001	4.180 [2.871, 6.086] < 0.001	3.986 [2.726, 5.828] < 0.001	3.396 [2.286, 5.045] < 0.001
*p* for trend	<0.001	<0.001	<0.001	<0.001
SIRI				
SIRI (per 1SD increase)	4.006 [3.216, 4.990] < 0.001	3.949 [3.159, 4.935] < 0.001	4.025 [3.181, 5.093] < 0.001	3.061 [2.392, 3.916] < 0.001
Tertiles of SIRI				
Tertile 1	Reference	Reference	Reference	Reference
Tertile 2	1.381 [0.942, 2.023] 0.098	1.248 [0.859, 1.813] 0.245	1.215 [0.835, 1.770] 0.309	1.278 [0.881, 1.854] 0.197
Tertile 3	2.604 [1.868, 3.631] < 0.001	2.659 [1.905, 3.711] < 0.001	2.409 [1.721, 3.373] < 0.001	2.104 [1.472, 3.006] < 0.001
*p* for trend	<0.001	<0.001	<0.001	<0.001

Model 1: no covariates were adjusted.

Model 2: age, sex, BMI, smoking status and drinking status were adjusted.

Model 3: Model 2 plus adjustment for SBP, DBP, TC, TG, HDL.C, LDL.C, and FPG.

Model 4: Model 3 plus adjustment for Statins, Antiplatelet medication, Beta-blockers, DM, Dyslipidemia and Hypertension.

AISI, aggregate index of systemic inflammation; CI, confidence interval; HR, hazard ratio; SII, Systemic Immune-Inflammation Index; SIRI, Systemic Inflammation Response Index.

Other abbreviations, see Table 1.

### 3.4 Dose–response relationship between inflammatory markers and the risk of stroke and its subtypes in patients with CHD

**Figure 5:**
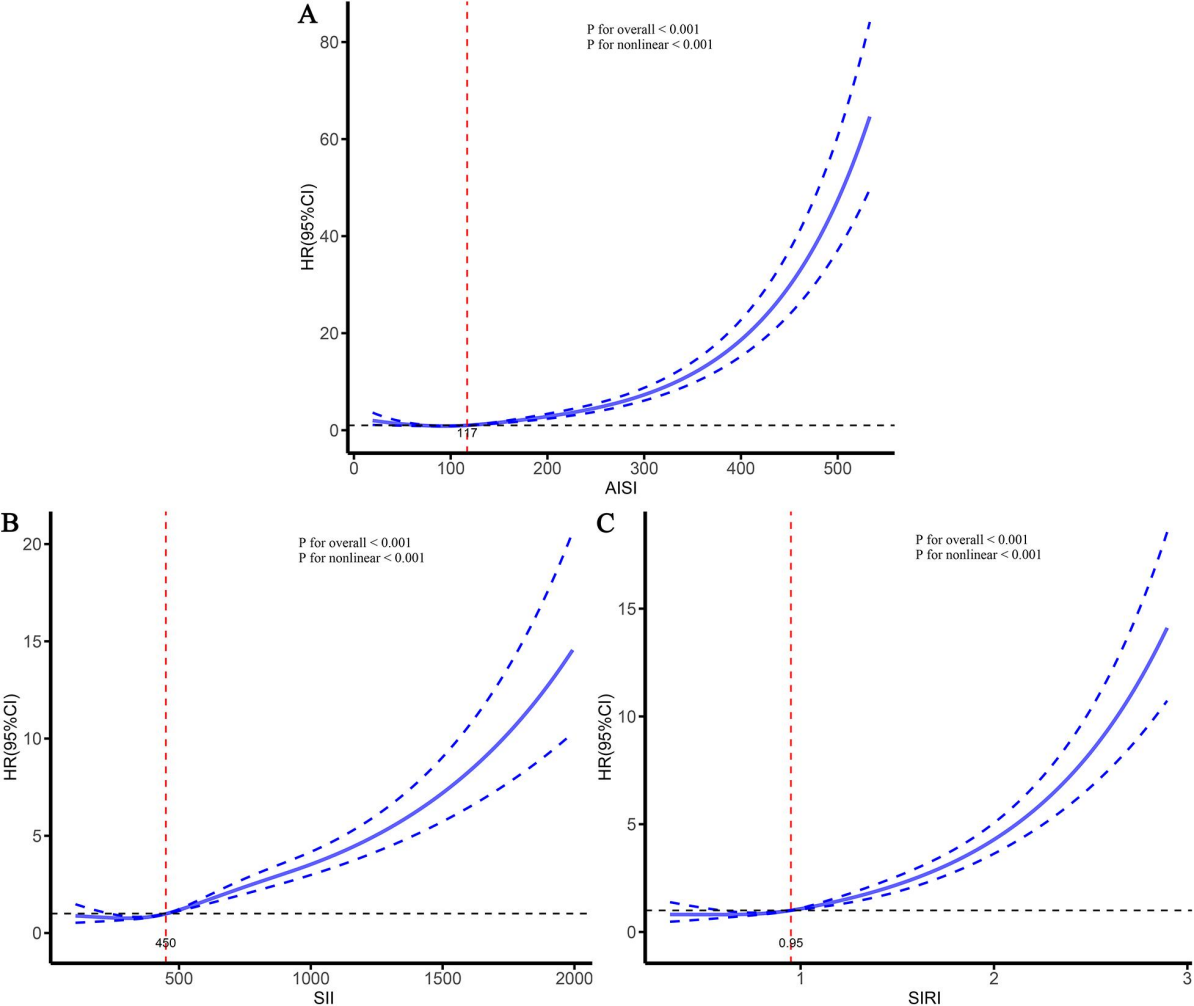
Dose–response relationships between three inflammatory markers and the risk of stroke. **(A)** AISI; **(B)** SII; **(C)** SIRI.

**Figure 6:**
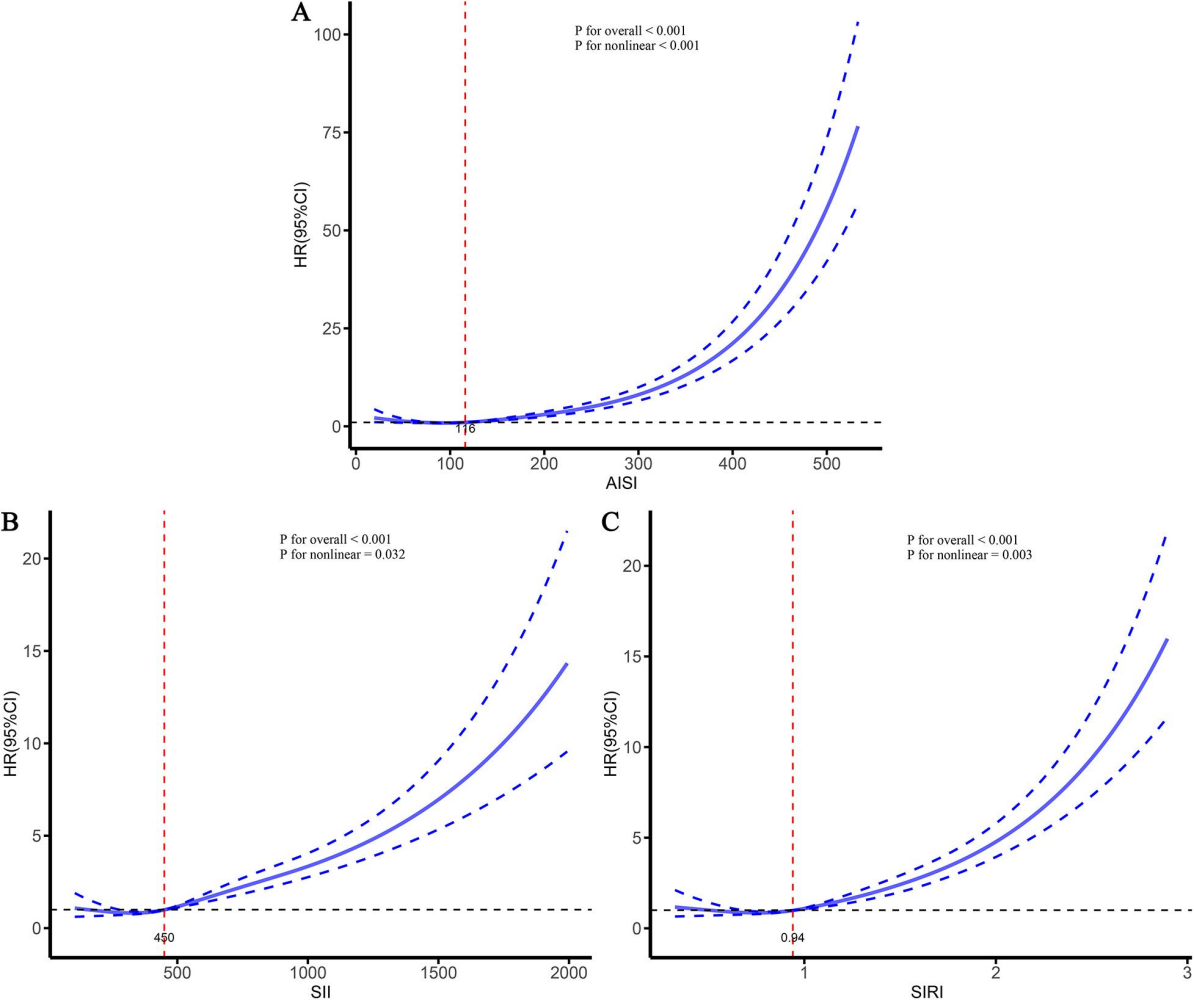
Dose–response relationships between three inflammatory markers and the risk of ischemic stroke. **(A)** AISI; **(B)** SII; **(C)** SIRI.

**Figure 7:**
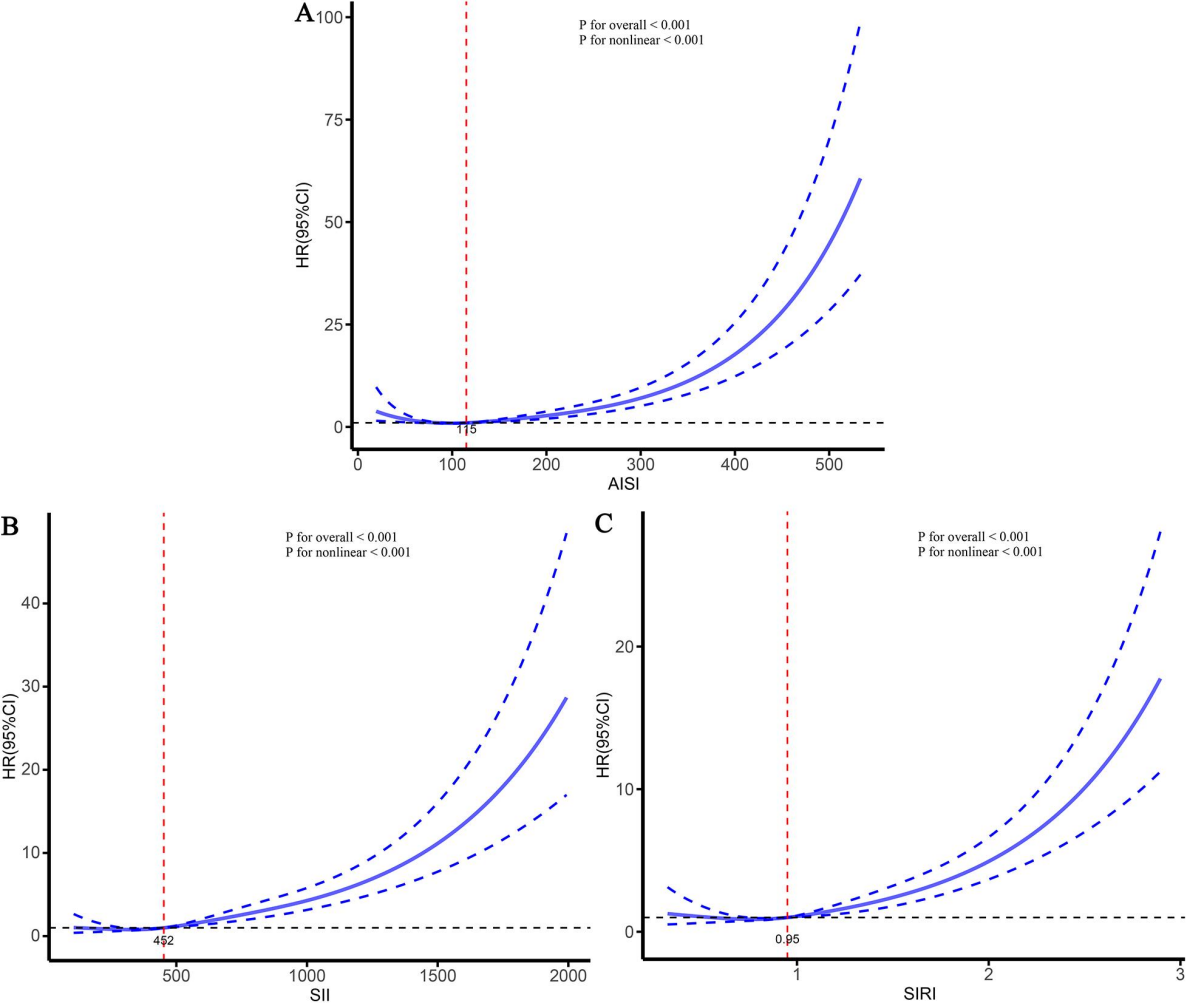
Dose–response relationships between three inflammatory markers and the risk of hemorrhagic stroke. **(A)** AISI; **(B)** SII; **(C)** SIRI.

To further characterize the dose–response relationships between the three inflammatory markers and the risks of stroke and its subtypes in patients with CHD, and to identify potential threshold effects, RCS analyses were performed (Figures 5–7). The analyses demonstrated a clear non-linear association between these inflammatory markers and the risk of overall stroke (*p* for nonlinear <0.001; Figure 5). Specifically, the risk of stroke increased markedly when AISI, SII, and SIRI values exceeded 117, 450, and 0.95, respectively. Consistent patterns were observed in analyses stratified by stroke subtype, in which the risks of both ischemic and hemorrhagic stroke rose significantly once marker levels surpassed their respective thresholds (Figures 6, 7).

### 3.5 Comparative analysis of the predictive performance of inflammatory markers for stroke and its subtypes in patients with CHD

**Figure 8:**
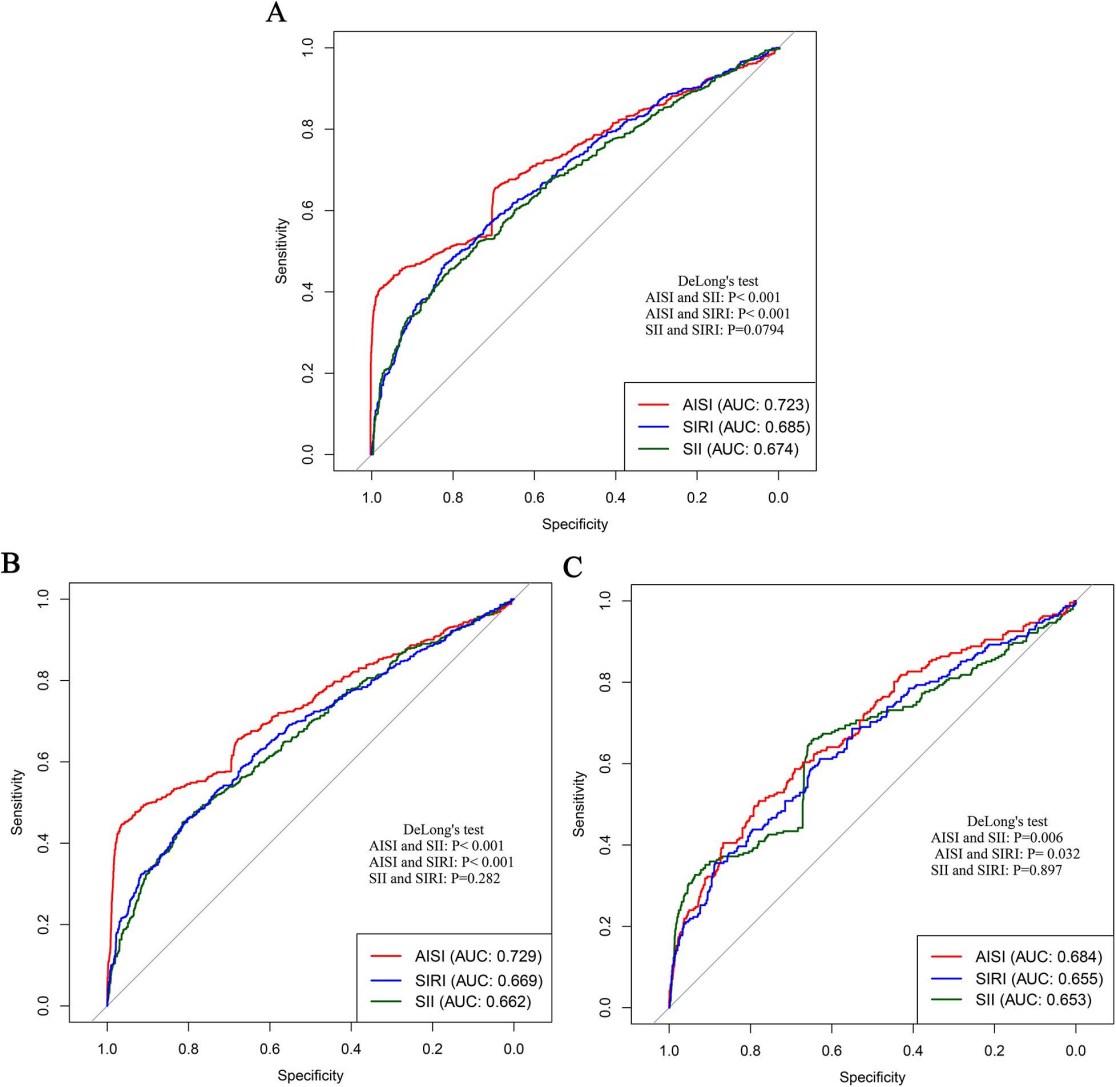
Comparison of the predictive performance of three inflammatory markers for stroke and its subtypes in patients with CHD. **(A)** Stroke; **(B)** Ischemic stroke; **(C)** Hemorrhagic stroke.

To compare the predictive performance of the three inflammatory markers for stroke and its subtypes in patients with CHD, a series of complementary analyses were conducted. First, the areas under the ROC curves were examined. The results showed that the area under the curve (AUC) values of AISI, SII, and SIRI for overall stroke were 0.723, 0.674, and 0.685, respectively. For ischemic stroke, the corresponding AUC values were 0.729, 0.662, and 0.669, whereas for hemorrhagic stroke they were 0.684, 0.653, and 0.655 (Table 4 and Figure 8). Collectively, these results indicated that AISI consistently demonstrated the best predictive performance.

**Table 4:** Comparative analysis was conducted on the ROC curves of various inflammatory biomarkers in predicting the risk of stroke and its subtypes in patients with CHD.

Inflammatory markers	AUC	95%CI low	95%CI up	Specificity	Sensitivity	Positive-pv	Negative-pv
Stroke							
AISI	0.723	0.701	0.745	0.977	0.408	0.753	0.904
SII	0.674	0.652	0.696	0.813	0.454	0.298	0.895
SIRI	0.685	0.664	0.707	0.817	0.471	0.309	0.899
Ischemic stroke							
AISI	0.729	0.703	0.755	0.965	0.446	0.612	0.934
SII	0.662	0.636	0.687	0.815	0.451	0.232	0.923
SIRI	0.669	0.644	0.695	0.803	0.460	0.224	0.923
Hemorrhagic stroke							
AISI	0.684	0.647	0.721	0.779	0.508	0.099	0.971
SII	0.653	0.613	0.694	0.650	0.657	0.083	0.975
SIRI	0.655	0.616	0.693	0.887	0.305	0.131	0.966

AISI, aggregate index of systemic inflammation; AUC, area under the curve; Negative-pv, negative predictive value; Positive-pv, positive predictive value; SII, Systemic Immune-Inflammation Index; SIRI, Systemic Inflammation Response Index.

Other abbreviations, see Table 1.

To further substantiate these results, C statistics were calculated. In the model 5, the addition of AISI, SII, and SIRI increased the C-index for overall stroke prediction to 0.908, 0.876, and 0.881, respectively, with AISI yielding the highest value. Similar patterns were observed in subtype analyses, where AISI achieved the highest C-index values for both ischemic stroke (0.912) and hemorrhagic stroke (0.868) (Table 5). Finally, validation analyses were performed using data from Huangshan City People's Hospital as an external verification cohort. Consistent with the primary analyses, both the C statistic and decision curve analysis demonstrated that AISI provided significantly better predictive performance than the other markers (Supplementary Table S5 and Figure S2). Overall, these results consistently support the conclusion that AISI is the most effective inflammatory marker for predicting the risk of stroke in patients with CHD.

**Table 5:** Comparative analysis of the predictive ability of various inflammatory indices in patients with CHD for the risk of stroke and its subtypes.

Inflammatory indices	C-index
Stroke	
Model 4	0.857
+AISI	0.908
+SII	0.876
+SIRI	0.881
Ischemic stroke	
Model 4	0.861
+AISI	0.912
+SII	0.875
+SIRI	0.882
Hemorrhagic stroke	
Model 4	0.828
+AISI	0.868
+SII	0.854
+SIRI	0.856

AISI, aggregate index of systemic inflammation; SII, Systemic Immune-Inflammation Index; SIRI, Systemic Inflammation Response Index.

Other abbreviations, see Table 1.

## 4 Discussion

Previous studies have consistently shown that inflammation is an important risk factor for CHD and stroke (28–30). However, existing inflammatory indicators are numerous and often lack comprehensiveness. Consequently, several new, simple, and easily accessible inflammatory markers have been developed, demonstrating improved predictive performance across various diseases (23, 27, 31). Given that studies on inflammation-related stroke risk among specific populations (such as patients with CHD) are relatively scarce, this study conducted a multicenter cohort analysis to deeply explore the relationship between various inflammatory markers and the risk of stroke and its subtypes in this population. Our study found that AISI, SII, and SIRI were significantly associated with stroke and its subtypes in patients with CHD. As the levels of these markers increased, the risk of stroke also gradually increased. The RCS curves further demonstrated a clear nonlinear dose-response relationship between AISI, SII, SIRI, and the risk of stroke and its subtypes. Threshold analyses indicated that when AISI, SII, and SIRI exceeded 117, 450, and 0.95, respectively, the risk of stroke increased significantly. Comparative analyses showed that all three markers performed well in predicting the occurrence of stroke and its subtypes; however, AISI, representing comprehensive inflammation, exhibited the strongest predictive performance. These findings suggest that AISI may hold greater clinical significance in assessing stroke risk among patients with CHD and highlight the potential benefit of maintaining inflammatory marker levels at relatively lower levels to reduce future stroke risk.

Current studies on the novel inflammatory markers AISI, SII, and SIRI have demonstrated their significant prognostic value across a range of diseases (23, 27, 31–33). In sepsis, these biomarkers—which integrate granulocyte, platelet, and lymphocyte counts—serve as powerful indicators of a dysregulated host response (34). Elevated levels of SII, SIRI, and AISI are closely associated with increased disease severity, organ dysfunction, and mortality, often outperforming traditional indicators such as C-reactive protein or white blood cell count (27, 34, 35). Similarly, in acute ischemic stroke, high levels of these markers at admission are independently associated with larger infarct volumes, more severe neurological deficits, and poorer functional outcomes at follow-up (32, 36). They are thought to reflect the degree of post-ischemic inflammation and the critical balance between pro-inflammatory and immunosuppressive states (36). In oncology, these indices have also shown strong predictive value (33, 37, 38). Elevated pre-treatment levels of SII, SIRI, and AISI consistently indicate lower overall survival and progression-free survival, reflecting the interplay between the pro-tumorigenic inflammatory milieu, thrombocytosis, and immune evasion mediated by lymphocytopenia (33, 38). Overall, these novel inflammatory markers are simple, easily accessible, and provide a comprehensive overview of systemic inflammation. Their predictive power and practicality make them valuable and cost-effective biomarkers for risk stratification and prognostic assessment in sepsis, stroke, and cancer.

The occurrence of stroke in patients with CHD due to inflammation is likely a complex process, as the pathogenesis of atherosclerosis itself involves the interaction of lipid peroxidation, immune activation, and chronic inflammation (39, 40). Several pathways may underlie inflammation-induced stroke. First, CHD can trigger lipid peroxidation, leading to an inflammatory response and vascular damage (41, 42). Some inflammatory cells and mediators promote the retention of low-density lipoprotein (LDL) particles beneath the endothelium, making them more susceptible to oxidative modification by reactive oxygen species (43–45). This generates oxidized LDL (ox-LDL), which contributes to the formation of cerebral thrombosis (46, 47). Second, inflammation can initiate a cascade of immune responses (48–50). Oxidized LDL acts as a damage-associated molecular pattern, promoting the recruitment and uncontrolled uptake of macrophages via phagocytic receptors, thereby forming foam cells—a hallmark of early fatty plaques (51, 52). This process is further amplified by adaptive immunity, including T cell activation and cytokine release (49). Finally, a self-sustaining inflammatory cascade occurs, characterized by the continuous secretion of pro-inflammatory cytokines and chemokines, which recruit additional white blood cells and proteases that degrade extracellular matrix proteins (53–55). This leads to plaque progression, expansion of the necrotic core, and ultimately plaque vulnerability and rupture, thereby triggering acute thrombotic complications (56, 57).

The main strengths of this study are as follows. First, it is based on a multi-center cohort design, covering a broad population, which enhances the reliability of the findings. Second, by employing a comprehensive statistical analysis system, this study is the first to demonstrate that several novel inflammatory markers are closely associated with an increased risk of stroke in patients with CHD. Additionally, comparative analyses identified AISI as the most predictive marker, a finding that has important clinical significance for risk assessment. Despite these strengths, several limitations should be acknowledged. First, although the study employed a multi-center design, all participants were Chinese male patients with CHD, which may limit the generalizability of the findings to other populations and ethnic groups. Second, the calculation of AISI, SII, and SIRI was based solely on baseline data and did not account for subsequent dynamic changes in these inflammatory markers. Future research should investigate the impact of these changes on stroke risk. Third, despite the statistical adjustments, the remaining confounding factors (which might stem from aspects such as the severity of CHD, clinical background, or the exact reasons for the non-use of certain drugs, etc., that are unmeasured) still cannot be completely eliminated. Fourth, the results of the *E*-value analysis indicate that these factors are very unlikely to overturn our findings. Fifth, our study lacks data on mortality. Future studies should also focus on exploring its impact on the risk of death. Finally, changes in patients' medication regimens or dietary habits during the follow-up period may have influenced inflammatory marker levels, and future studies should take these factors into consideration.

## 5 Conclusion

This study is the first to systematically demonstrate that the three novel inflammatory markers AISI, SII, and SIRI are closely associated with an increased risk of stroke and its subtypes in patients with CHD, exhibiting excellent predictive performance. Among them, AISI was identified as the marker with the highest predictive value. As a feasible and easily obtainable indicator, AISI may have significant clinical utility for assessing stroke risk in patients with CHD and for guiding early interventions to maintain inflammation at lower levels.

## Data Availability

The original contributions presented in the study are included in the article/Supplementary Material, further inquiries can be directed to the corresponding author.
