# Supplementary material for: Elevated inflammatory index and future risk of stroke in patients with coronary heart disease: a multicenter prospective cohort study
Source: Front Cardiovasc Med. 2026 Jan 12;12:1755408. doi: 10.3389/fcvm.2025.1755408 (PMC12832916; doi:10.3389/fcvm.2025.1755408)
Supplement: Supplementary file 1 [file Datasheet1.docx]

Supplementary Material

# Supplemental material and methods

**Baseline examination**

Anthropometric measurements were taken by trained nurses. Data for height and weight were 3 acquired following a protocol standardized to an accuracy of 0.1 kg and 0.1 cm, respectively. Current smokers were defined as having smoked 100 cigarettes in their lifetime and currently smoking. Alcohol consumption was evaluated with questions regarding the types of alcoholic beverages, the frequency of alcohol consumption per week, and the usual amount consumed per occasion. Subjects who reported alcohol consumption >140 g/week for men and >70 g/week for women were deemed to have excessive alcohol consumption (1).

**Definitions**

Criteria for hypertension included self-reported hypertension, current use of anti-hypertensive medication, or systolic blood pressure (SBP) ≥ 140 mmHg and/or diastolic blood pressure (DBP) ≥ 90 mmHg recorded for at least three consecutive readings. diabetes was defined as fasting serum glucose ≥7.0 mmol/L, the 2-h serum glucose of the oral glucose tolerance test ≥11.1 mmol/L, or the current use of hypoglycaemic medication or insulin. coronary heart disease (CHD) was defined as a fatal or nonfatal myocardial infarction, unstable angina, and coronary revascularization. Hyperlipidemia is defined as an abnormal elevation of lipid levels in the bloodstream, primarily characterized by increased concentrations of total cholesterol (TC), low-density lipoprotein cholesterol (LDL-C), and triglycerides (TG), or a reduction in high-density lipoprotein cholesterol (HDL-C). According to international guidelines, hyperlipidemia is diagnosed when TC levels exceed 6.2 mmol/L (240 mg/dL), LDL-C levels are ≥ 4.1 mmol/L (160 mg/dL), TG levels are ≥ 2.3 mmol/L (200 mg/dL), or HDL-C levels fall below 1.0 mmol/L (40 mg/dL) in men and 1.3 mmol/L (50 mg/dL) in women.

Ischemic and hemorrhagic strokes were defined and classified based on the World Health Organization (WHO) criteria, supplemented by neuroimaging confirmation (computed tomography [CT] or magnetic resonance imaging [MRI]). Specifically:

1. **Ischemic stroke** was defined as rapidly developing clinical signs of focal (or global) cerebral dysfunction lasting more than 24 hours or leading to death, with no apparent cause other than vascular origin, and with neuroimaging evidence of cerebral infarction.

2. **Hemorrhagic stroke** was defined as rapidly developing clinical signs of neurological dysfunction attributable to a focal collection of blood within the brain parenchyma or ventricular system (intracerebral hemorrhage) or subarachnoid space (subarachnoid hemorrhage), as confirmed by neuroimaging.

All stroke events were adjudicated by a clinical endpoint committee consisting of experienced neurologists and cardiologists who reviewed the medical records and neuroimaging reports.

The body mass index (BMI) was calculated as per the formula: Weight (kg)/Height^2^ (m).

**Details of the statistical analyses**.

Variables of baseline characteristics are shown as n (%) if categorical, mean (SD) if normally distributed, and median (interquartile range) if nonnormally distributed. To compare the characteristics among different inflammatory markers groups, the chi-square test was performed for categorical variables, and one-way analysis of variance, or the Kruskal-Wallis test, was performed for continuous variables with normal and skewed distributions.

The association between inflammatory index and future risk of stroke in patients with coronary heart disease was tested with multivariable Cox regression models. This study set four different models (Model 1: unadjusted; Model 2: age, sex, BMI, smoking status and drinking status were adjusted; Model 3: Model 2 plus adjustment for SBP, DBP, TC, TG, HDL.C, LDL.C, and FPG. Model 4: Model 3 plus adjustment for Statins, Antiplatelet medication, Beta-blockers, DM, Dyslipidemia and Hypertension.) to adjust. Tests for trend were conducted, assigning the median value within each tertile to the corresponding tertile. In addition, the restricted inverse square spline (four nodes at the 5th, 35th, 65th, and 95th percentiles of the inflammatory markers distribution) was used to evaluate the nonlinear relationships. Finally, receiver operating characteristic (ROC) curves and C statistics were applied to compare the predictive performance of these markers.

All analyses were done using R (version 4.4.3). All P-values were two-sided, and P-values of <0.05 denoted statistical significance.

1. **Supplementary Tables**

**Table S1.** Covariance Diagnostics

| Variable | VIF |
| --- | --- |
| Age | 3.146775 |
| Sex | 2.002386 |
| BMI | 1.128773 |
| SBP | 1.740032 |
| DBP | 1.816712 |
| smoking status | 1.573644 |
| drinking status | 3.456508 |
| TC | 1.424347 |
| TG | 1.527557 |
| HDL.C | 1.589715 |
| LDL.C | 1.004766 |
| FPG | 1.256333 |
| Dyslipidemia | 1.127536 |
| Hypertension | 1.006599 |
| DM | 2.728855 |
| Statins | 1.151215 |
| Antiplatelet medication | 1.164649 |
| Beta-blockers | 1.111626 |

VIF = 1/(1-R^2^). VIF step-by-step screening method: Calculate the VIF of each variable. If the maximum VIF value is ≥ 10, remove the variable with the maximum VIF value.

VIF: variance inflation factors.

**Table S2.** Relationship between inflammatory indicators in patients with CHD and stroke in patients of Huangshan City People's Hospital

| **Stroke** | Model 1 | Model 2 | Model 3 | Model 4 |
| --- | --- | --- | --- | --- |
|  | HR (95% CI) P | HR (95% CI) P | HR (95% CI) P | HR (95% CI) P |
| AISI |  |  |  |  |
| AISI (per 1SD increase) | 2.337 [2.181, 2.505] <0.001 | 2.188 [2.035, 2.352] <0.001 | 2.164 [2.006, 2.335] <0.001 | 2.177 [2.001, 2.369] <0.001 |
| Tertiles of AISI |  |  |  |  |
| Tertile 1 | Reference | Reference | Reference | Reference |
| Tertile 2 | 2.398 [1.933, 2.975] <0.001 | 2.311 [1.861, 2.869] <0.001 | 2.302 [1.853, 2.861] <0.001 | 3.077 [2.439, 3.881] <0.001 |
| Tertile 3 | 4.552 [3.740, 5.539] <0.001 | 4.264 [3.499, 5.195] <0.001 | 3.898 [3.193, 4.759] <0.001 | 5.439 [4.381, 6.751] <0.001 |
| P for trend | <0.001 | <0.001 | <0.001 | <0.001 |
| SII |  |  |  |  |
| SII (per 1SD increase) | 2.385 [2.216, 2.567] <0.001 | 2.236 [2.069, 2.418] <0.001 | 2.257 [2.080, 2.448] <0.001 | 2.011 [1.848, 2.189] <0.001 |
| Tertiles of SII |  |  |  |  |
| Tertile 1 | Reference | Reference | Reference | Reference |
| Tertile 2 | 2.431 [1.956, 3.021] <0.001 | 2.420 [1.942, 3.015] <0.001 | 2.330 [1.868, 2.905] <0.001 | 3.013 [2.403, 3.777] <0.001 |
| Tertile 3 | 7.081 [5.846, 8.575] <0.001 | 6.644 [5.472, 8.066] <0.001 | 6.039 [4.962, 7.349] <0.001 | 5.541 [4.520, 6.794] <0.001 |
| P for trend | <0.001 | <0.001 | <0.001 | <0.001 |
| SIRI |  |  |  |  |
| SIRI (per 1SD increase) | 3.469 [3.098, 3.884] <0.001 | 3.226 [2.867, 3.629] <0.001 | 3.084 [2.734, 3.479] <0.001 | 2.723 [2.406, 3.083] <0.001 |
| Tertiles of SIRI |  |  |  |  |
| Tertile 1 | Reference | Reference | Reference | Reference |
| Tertile 2 | 1.896 [1.542, 2.333] <0.001 | 1.742 [1.411, 2.150] <0.001 | 1.642 [1.329, 2.028]] <0.001 | 1.991 [1.604, 2.471] <0.001 |
| Tertile 3 | 5.006 [4.159, 6.024] <0.001 | 4.546 [3.767, 5.487] <0.001 | 4.143 [3.427, 5.007] <0.001 | 4.440 [3.635, 5.424] <0.001 |
| P for trend | <0.001 | <0.001 | <0.001 | <0.001 |

Model 1: no covariates were adjusted.

Model 2: age, sex, BMI, smoking status and drinking status were adjusted.

Model 3: Model 2 plus adjustment for SBP, DBP, TC, TG, HDL.C, LDL.C, and FPG.

Model 4: Model 3 plus adjustment for Statins, Antiplatelet medication, Beta-blockers, DM, Dyslipidemia and Hypertension..

Abbreviations: AISI, aggregate index of systemic inflammation; SII, Systemic Immune-Inflammation Index; SIRI, Systemic Inflammation Response Index; HR, hazard ratio; CI, confidence interval

Other abbreviations, see Table 1.

**Table S3.** Relationship between inflammatory indicators in patients with CHD and the subtypes of stroke in patients of Huangshan City People's Hospital

| **Stroke subtype** | Model 1 | Model 2 | Model 3 | Model 4 |
| --- | --- | --- | --- | --- |
|  | HR (95% CI) P | HR (95% CI) P | HR (95% CI) P | HR (95% CI) P |
| **Ischemic stroke** |  |  |  |  |
| **AISI** |  |  |  |  |
| AISI (per 1SD increase) | 2.447 [2.262, 2.648] <0.001 | 2.243 [2.066, 2.436] <0.001 | 2.230 [2.046, 2.430] <0.001 | 2.341 [2.126, 2.578] <0.001 |
| Tertiles of AISI |  |  |  |  |
| Tertile 1 | Reference | Reference | Reference | Reference |
| Tertile 2 | 2.301 [1.776, 2.982] <0.001 | 2.222 [1.714, 2.881] <0.001 | 2.229 [1.717, 2.893] <0.001 | 3.199 [2.426, 4.217] <0.001 |
| Tertile 3 | 5.070 [4.027, 6.383] <0.001 | 4.735 [3.756, 5.968] <0.001 | 4.371 [3.460, 5.522] <0.001 | 6.524 [5.069, 8.396]  <0.001 |
| P for trend | <0.001 | <0.001 | <0.001 | <0.001 |
| **SII** |  |  |  |  |
| SII (per 1SD increase) | 2.334 [2.140, 2.545] <0.001 | 2.138 [1.953, 2.341] <0.001 | 2.160 [1.965, 2.374] <0.001 | 1.950 [1.768, 2.150] <0.001 |
| Tertiles of SII |  |  |  |  |
| Tertile 1 | Reference | Reference | Reference | Reference |
| Tertile 2 | 2.300 [1.803, 2.933] <0.001 | 2.261 [1.767, 2.894] <0.001 | 2.196 [1.714, 2.813] <0.001 | 2.886 [2.240, 3.720] <0.001 |
| Tertile 3 | 6.073 [4.894, 7.535] <0.001 | 5.702 [4.581, 7.096]  <0.001 | 5.242 [4.200, 6.541] <0.001 | 4.938 [3.921, 6.219] <0.001 |
| P for trend | <0.001 | <0.001 | <0.001 | <0.001 |
| **SIRI** |  |  |  |  |
| SIRI (per 1SD increase) | 3.534 [3.102, 4.025] <0.001 | 3.206 [2.798, 3.672] <0.001 | 3.087 [2.687, 3.546] <0.001 | 2.806 [2.434, 3.236] <0.001 |
| Tertiles of SIRI |  |  |  |  |
| Tertile 1 | Reference | Reference | Reference | Reference |
| Tertile 2 | 1.784 [1.400, 2.273] <0.001 | 1.625 [1.270, 2.079] <0.001 | 1.546 [1.207, 1.980]  <0.001 | 1.878 [1.460, 2.415] <0.001 |
| Tertile 3 | 5.023 [4.056, 6.220] <0.001 | 4.497 [3.619, 5.587] <0.001 | 4.114 [3.306, 5.120] <0.001 | 4.593 [3.647, 5.786] <0.001 |
| P for trend | <0.001 | <0.001 | <0.001 | <0.001 |
| **Hemorrhagic stroke** |  |  |  |  |
| **AISI** |  |  |  |  |
| AISI (per 1SD increase) | 2.182 [1.909, 2.494] <0.001 | 2.167 [1.884, 2.493] <0.001 | 2.134 [1.838, 2.477 <0.001 | 1.925 [1.636, 2.267] <0.001 |
| Tertiles of AISI |  |  |  |  |
| Tertile 1 | Reference | Reference | Reference | Reference |
| Tertile 2 | 1.996 [1.395, 2.857] <0.001 | 1.946 [1.357, 2.791] <0.001 | 1.867 [1.299, 2.683] <0.001 | 1.813 [1.206, 2.724] 0.004 |
| Tertile 3 | 2.945 [2.094, 4.141] <0.001 | 2.882 [2.042, 4.067] <0.001 | 2.517 [1.777, 3.566] <0.001 | 2.643 [1.791, 3.898] <0.001 |
| P for trend | <0.001 | <0.001 | <0.001 | <0.001 |
| **SII** |  |  |  |  |
| SII (per 1SD increase) | 2.695 [2.374, 3.059] <0.001 | 2.724 [2.368, 3.133] <0.001 | 2.771 [2.394, 3.207] <0.001 | 2.397 [2.054, 2.797] <0.001 |
| Tertiles of SII |  |  |  |  |
| Tertile 1 | Reference | Reference | Reference | Reference |
| Tertile 2 | 2.889 [1.897, 4.400] <0.001 | 2.910 [1.903, 4.450]  <0.001 | 2.658 [1.734, 4.076] <0.001 | 3.223 [2.080, 4.994] <0.001 |
| Tertile 3 | 8.589 [5.893, 12.519]  <0.001 | 8.259 [5.644, 12.085]  <0.001 | 7.157 [4.867, 10.526]  <0.001 | 6.028 [4.044, 8.985]  <0.001 |
| P for trend | <0.001 | <0.001 | <0.001 | <0.001 |
| **SIRI** |  |  |  |  |
| SIRI (per 1SD increase) | 3.761 [3.057, 4.625] <0.001 | 3.803 [3.063, 4.721] <0.001 | 3.590 [2.878, 4.479] <0.001 | 2.940 [2.338, 3.698] <0.001 |
| Tertiles of SIRI |  |  |  |  |
| Tertile 1 | Reference | Reference | Reference | Reference |
| Tertile 2 | 1.866 [1.286, 2.708] 0.001 | 1.828 [1.252, 2.668] 0.002 | 1.629 [1.113, 2.383]  0.012 | 1.836 [1.237, 2.726] 0.003 |
| Tertile 3 | 4.515 [3.221, 6.329] <0.001 | 4.462 [3.163, 6.292] <0.001 | 3.932 [2.780, 5.563] <0.001 | 3.699 [2.561, 5.342] <0.001 |
| P for trend | <0.001 | <0.001 | <0.001 | <0.001 |

Model 1: no covariates were adjusted.

Model 2: age, sex, BMI, smoking status and drinking status were adjusted.

Model 3: Model 2 plus adjustment for SBP, DBP, TC, TG, HDL.C, LDL.C, and FPG.

Model 4: Model 3 plus adjustment for Statins, Antiplatelet medication, Beta-blockers, DM, Dyslipidemia and Hypertension..

Abbreviations: AISI, aggregate index of systemic inflammation; SII, Systemic Immune-Inflammation Index; SIRI, Systemic Inflammation Response Index; HR, hazard ratio; CI, confidence interval

Other abbreviations, see Table 1.

Table S4 E value for the association between inflammatory indicators in patients with coronary heart disease and stroke as well as stroke subtypes.

|  | Stroke | Ischemic stroke | Hemorrhagic stroke |
| --- | --- | --- | --- |
| AISI (per 1SD increase) |  |  |  |
| E-value for point estimate | 5.674 | 5.673 | 4.761 |
| SII (per 1SD increase) |  |  |  |
| E-value for point estimate | 3.429 | 3.305 | 4.410 |
| SIRI (per 1SD increase) |  |  |  |
| E-value for point estimate | 5.079 | 5.324 | 5.573 |

The observed associations are the fully adjusted hazard ratios (95% confidence intervals) shown in Tables 2 and 3 and are presented here for reference..

Table S5. A comparative analysis of the predictive ability of various inflammatory indicators in patients with coronary heart disease at Huangshan City People's Hospital and their association with stroke risk and its subtypes

| Inflammatory indices | C-index |
| --- | --- |
| **Stroke** | |
| Model 4 | 0.852 |
| +AISI | 0.902 |
| +SII | 0.870 |
| +SIRI | 0.874 |
| **Ischemic stroke** | |
| Model 4 | 0.853 |
| +AISI | 0.908 |
| +SII | 0.871 |
| +SIRI | 0.876 |
| **Hemorrhagic stroke** | |
| Model 4 | 0.821 |
| +AISI | 0.867 |
| +SII | 0.849 |
| +SIRI | 0.854 |

Abbreviations: AISI, aggregate index of systemic inflammation; SII, Systemic Immune-Inflammation Index; SIRI, Systemic Inflammation Response Index

Other abbreviations, see Table 1.

1. **Supplementary Figure**

**
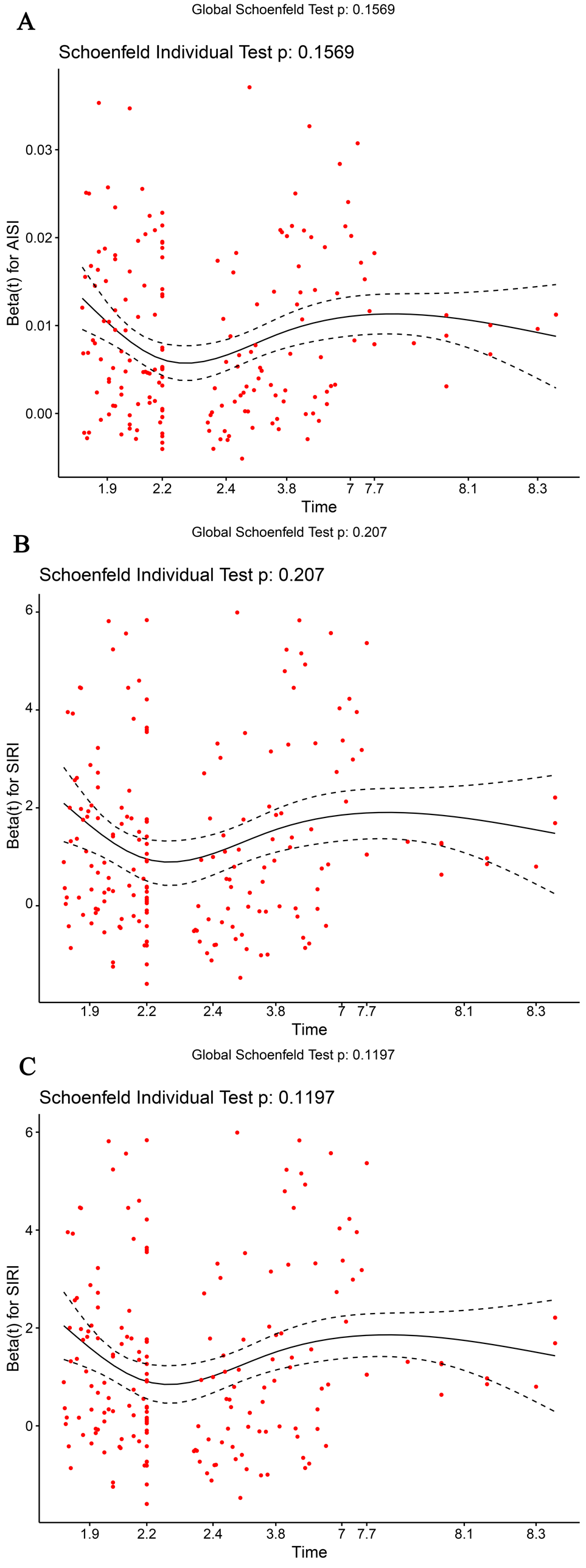
**

**Figure S1 Proportional hazards assumption**

**A, AISI; B, SII; C, SIRI**

**

**

**Figure S2 Decision curve analysis of different validation indicators for the predictive performance of stroke and its subtypes**

**(A), Stroke; (B), Ischemic stroke; (C), Hemorrhagic stroke**

**References**

1. Farrell GC, Chitturi S, Lau GK, Sollano JD. Guidelines for the assessment and management of non-alcoholic fatty liver disease in the Asia-Pacific region: executive summary. Journal of gastroenterology and hepatology. 2007;22(6):775-7. Epub 2007/06/15. doi: 10.1111/j.1440-1746.2007.05002.x. PubMed PMID: 17565629.
